# Status of Retinoids and Carotenoids and Associations with Clinical Outcomes in Maternal-Infant Pairs in Nigeria

**DOI:** 10.3390/nu10091286

**Published:** 2018-09-12

**Authors:** Corrine Hanson, Elizabeth Lyden, Ann Anderson-Berry, Nicholas Kocmich, Amy Rezac, Shirley Delair, Jeremy Furtado, Matthew Van Ormer, NI Izevbigie, EK Olateju, GA Adaba, EA Anigilaje, Tahiru Tahiru, Stephen Obaro

**Affiliations:** 1 College of Allied Health Professions Medical Nutrition Education, University of Nebraska Medical Center, Omaha, NE 68198-4045, USA; 2 College of Public Health, University of Nebraska Medical Center, Omaha, NE 68198-4375, USA; elyden@unmc.edu; 3 Pediatrics 981205 Nebraska Medical Center, University of Nebraska Medical Center, Omaha, NE 68198-1205, USA; alanders@unmc.edu (A.A.-B.); nicholas.kocmich@unmc.edu (N.K.); amy.rezac@unmc.edu (A.R.); shirley.delair@unmc.edu (S.D.); matthew.vanormer@unmc.edu (M.V.O.); stephen.obaro@unmc.edu (S.O.); 4 Department of Nutrition, Harvard School of Public Health 655 Huntington Avenue, Boston, MA 02215, USA; jfurtado@hsph.harvard.edu; 5 University of Abuja Teaching Hospital Gwagwalada-Zuba, Gwagwalada P.M.B. 228, Nigeria; sakharemog@yahoo.co.uk (N.I.I.); oeyinade@yahoo.com (E.K.O.); docakabago@yahoo.com (G.A.A.); naijanannytales@yahoo.com (E.A.A.); tahiroshma@yahoo.com (T.T.)

**Keywords:** vitamin A, carotenoids, lutein, β-carotene, pregnancy, maternal-child

## Abstract

Vitamin A is an essential nutrient in pregnancy, and other carotenoids have been independently associated with maternal-infant outcomes. The objective of this study was to quantify the status of vitamin A and carotenoids in Nigerian maternal-infant pairs at delivery, compare these to a cohort from a developed nation, and determine the impact on clinical outcomes. Maternal and cord blood samples were collected in 99 Nigerian mother-infant pairs. Concentrations of lutein + zeaxanthin, β-cryptoxanthin, lycopene, α- and β-carotenes, and retinol were measured using HPLC. Descriptive statistics were calculated and Spearman coefficients were used to assess correlations between maternal and cord measurements; Mann-Whitney tests were used to compare median plasma values between dichotomous variables. Linear regression models were used to adjust for relevant confounders. A *p* < 0.05 was considered statistically significant. Thirty-five percent of mothers had plasma retinol concentrations ≤0.70 µmol/L; 82% of infants had plasma retinol concentrations ≤0.70 µmol/L at delivery. Maternal and infant concentrations of vitamin A compounds were highly correlated and were associated with newborn growth and Apgar scores. Despite plasma concentrations of pro-vitamin A carotenoids higher than those reported in other populations, pregnant Nigerian women have a high prevalence of vitamin A deficiency. As vitamin A related compounds are modifiable by diet, future research determining the clinical impact of these compounds is warranted.

## 1. Introduction

Vitamin A is an essential nutrient throughout the life cycle; however, its influence is particularly critical during periods when cells are rapidly proliferating and differentiating, including during the fetal and newborn periods [1]. Vitamin A deficiency (VAD) is one of the most prevalent and important nutritional deficiencies worldwide, and is known to have been a public health threat in developing countries for several decades [2]. Nearly 10 million women, mostly in underdeveloped nations, suffer from night blindness during pregnancy [3], a condition that is often caused by vitamin A deficiency, and this is also associated with an assemblage of other adverse health outcomes and nutritional considerations in both mothers and their infants [4,5,6]. Dietary vitamin A is found as preformed retinol in animal products, such as eggs, liver and milk, and as retinyl esters in fortified foods [7]. Vitamin A supplementation during pregnancy has been found to improve hematological status and improve maternal anemia [3,8]; however, some evidence also suggests vitamin A supplementation should be used with caution in HIV-positive mothers [8], and in high doses can be teratogenic to the developing fetus [9]. Serum retinol concentrations have been widely used and are recommended as the prime indicator for routine assessment of the occurrence and degree of vitamin A deficiency; however, studies of animals fed a low-retinol diet show that liver stores of retinol decline to marginal status before serum retinol levels begin to decrease. However, given the invasiveness of liver biopsies to asses liver stores, and the time and resources needed for other measures of vitamin A status such as the relative dose-response test, serum retinol concentrations are commonly used as a marker of vitamin A status in population-based studies. Although there is no formal definition of vitamin A status during pregnancy, multiple other studies and the World Health Organization have defined vitamin A sufficiency during pregnancy as ˃1.05 µmol/L and deficiency as ≤0.70 [5]. The prevalence of individuals with serum retinol ≤0.70 /L µmol is used by the World Health Organization to assess population VAD and to reflect the severity of VAD as a public health problem [1].

Vitamin A-related compounds such as carotenoids may have important biological properties in a newborn [10] and unique roles in eye and brain development independent of retinol [10,11]. Biologically significant carotenoids include α-carotene, β-carotene, and β-cryptoxanthin, which have pro-vitamin A activity. Lycopene and lutein, while not possessing pro-vitamin A activity, are effective anti-oxidants [12,13,14]. Carotenoids are found primarily in foods of plant origin, including leafy green and red, yellow, and orange fruits and vegetables [12,15]. Because dietary carotenoid intake patterns vary around the world depending on the availability of specific fruits and vegetables, and because the overall health and nutritive status of women in the U.S. differs from that of women in developing parts of the world, it is important to describe the status of carotenoids in maternal and cord plasma in different populations. While supplementation β-carotene during pregnancy has been studied, with mixed results [16,17,18,19], little is known about the status of other carotenoids in diverse populations of pregnant women, or their associations with clinical outcomes.

The purpose of this study was to compare status of retinoids and carotenoids and associated pregnancy outcomes between maternal infant dyads in a developed and a developing nation to identify potentially modifiable differences that may affect pregnancy and neonatal outcomes and provide a way to improve maternal and neonatal health.

## 2. Materials and Methods

### 2.1. Recruitment

The goal of this study was to compare retinol and carotenoid status in pregnant women and their infants at the time of delivery in an underdeveloped nation (Nigeria) and a developed nation (U.S.). Nigerian subjects were enrolled while attending the antenatal clinic or delivery center at the University of Abuja Teaching Hospital in Gwagwalada, Nigeria. U.S. subjects were recruited from the Nebraska Medicine Labor and Delivery unit, Newborn Nursery, and the Newborn Intensive Care unit in Omaha, Nebraska, United States, an academic medical center serving a delivery population with racial demographics that closely mirror the racial demography of the United States population.

### 2.2. Ethical Approvals

Ethical approval for the Nigerian cohort was granted from University of Abuja Teaching Hospital, Gwagwalada in Abuja, Nigeria. Separate IRB approval was obtained from the University of Nebraska Medical Center for participant enrollment in the United States.

### 2.3. Sample and Data Collection

Samples of both cord and maternal blood were collected at the time of delivery from those who consented to participate. Relevant clinical data was collected from both populations. Inclusion criteria for the Nigerian population included maternal age ≥18 years, gestational age ≥24 weeks, infant age 0–7 days at enrollment (however all mother/infant dyads were enrolled at or before delivery), absence of heavy peripartal vaginal bleeding, and ability to provide written informed consent. Exclusion criteria for the U.S. population included congenital abnormalities, gastroinstestinal, liver, or kidney disease, or inborn errors of metabolism in the infant or the mother. Demographic and clinical data from the Nigerian subjects was prospectively collected and entered into electronic capture tools, collectively termed REDCap. As part of this data collection, a 28-day follow-up phone interview was conducted with participating Nigerian mothers to assess infant well-being and interval illness or hospitalization. Pertinent demographic and clinical data was collected from the U.S. population’s electronic medical records. Clinical data collected from both populations included maternal age, body mass index (BMI), smoking status, infant corrected gestational age, birth anthropometrics, gender, and Apgar scores (1-min. and 5-min).

### 2.4. Biochemical Analysis

Blood samples were protected from heat and light, processed, and frozen at −80 °C within a maximum of 12 h. Analysis of samples was performed at the Biomarker Research Institute, Harvard School of Public Health. Concentrations of total retinoids and carotenoids in plasma were measured as described by El-Sohemy et al., who found plasma levels to correlate well with fat tissue samples using this method [20]. Plasma samples were mixed with ethanol containing 10 mg/mL rac-Tocopherol (Tocol) as an internal standard, extracted with hexane, evaporated to dryness under nitrogen, and reconstituted in ethanol-dioxane (1:1 *v/v*) and acetonitrile. Samples were quantitated by high-performance liquid chromatography (HPLC) on a Restek Ultra C18 150 mm × 4.6 mm column, 3mm particle size encased in a column oven (Hitachi L-2350, Hitachi, San Jose, CA, USA) to prevent temperature fluctuations, and equipped with a trident guard cartridge system (Restek, Corp. Bellefonte, PA, USA). A mixture of acetonitrile, tetrahydrofuran, methanol, and a 1% ammonium acetate solution (68:22:7:3) was used as mobile phase at a flow rate of 1.1 mL/min, with a Hitachi L-2130 pump in isocratic mode, a Hitachi L-2455 diode array detector (300 nm and 445 nm), and a Hitachi L-2200 auto-sampler with water-chilled tray. The Hitachi System Manager software (D-2000 Elite, Version 3.0, Hitachi, San Jose, CA, USA) was used for peak integration and data acquisition. Internal quality control was monitored with four control samples analyzed within each run. These samples consist of two identical high-level plasmas and two identical low-level plasmas. Comparison of data from these samples allows within-run and between-run variation estimates. In addition, external quality control was monitored by participation in the standardization program for carotenoid analysis from the National Institute of Standards and Technology USA

Multiple other studies and the World Health Organization have defined vitamin A sufficiency during pregnancy as ˃1.05 µmol/L and deficiency as ≤0.70 [5,21,22,23,24]. Therefore, we used the following retinol categories to classify the vitamin A status of our population: severely deficient ≤0.35 µmol/L; deficient ˃0.35–0.70 µmol/L; insufficient ˃0.70–1.05 µmol/L; and adequate ˃1.05 µmol/L. These categories were also applied to the infants in our study [21].

### 2.5. Growth Outcomes

Birth growth parameter percentiles were constructed for infant birth weight, length, and head circumference using international standards from the INTERGROWTH-21st Project. Birth growth parameter z-scores were constructed using the WHO Child Growth Parameter’s Anthro software for SPSS.

### 2.6. Statistical Analysis

Data was summarized using means, standard deviations, medians, ranges, counts, and percentages. Spearman correlation coefficients were used to look at the association of maternal and cord blood measurements, as well as correlation of plasma tocopherol levels with select clinical outcomes. Demographic variables were compared using independent sample *t*-test or Fisher’s exact test. The Mann-Whitney test was used to compare median plasma values between dichotomous variables. Normally distributed data is presents as means (SD), while non-normally distributed data is presented as median (IQR). Statistical significance was set at *p* ≤ 0.05. SAS version 9.4 (SAS Institute, Cary, NC, USA) was used for all analysis.

## 3. Results

### 3.1. Baseline Characteristics

Ninety-nine Nigerian and 179 United States maternal-infant pairs were included in the study. In comparison to the U.S. population, the Nigerian population was slightly older with a higher pre-pregnancy BMI, and had a lower overall percentage of smokers. In the Nigerian cohort, 87 mothers and 74 infants had plasma available for analysis; of the U.S. subjects, 176 mothers and 167 infants had blood samples analyzed at the time of delivery. Demographic characteristics of both populations are shown in Table 1. Results of clinical outcomes and associations with vitamin A and carotenoids within the U.S. population have been published elsewhere [25], and in this manuscript, results of the U.S. cohort are limited to a comparison of nutrient status with the Nigeria cohort.

### 3.2. Plasma Retinol Results

Overall, the median plasma retinol concentration of the Nigerian maternal population was 0.81 µmol/L (IQR: 0.43 µmol/L), while the median plasma newborn concentration was 0.49 µmol/L (IQR: 0.19 µmol/L). Thirty-five percent of mothers met the criteria for vitamin A deficiency and an additional 40% were insufficient. One-quarter (25%) had adequate plasma retinol concentrations. Nearly all infants were either insufficient, deficient, or severely deficient: 16.2% had insufficient concentrations, 67.6% had deficient concentrations, and 14.7% had severely deficient concentrations; only 1.3% of infants were adequate in vitamin A. Table 2 shows the prevalence of vitamin A insufficiency, deficiency and severe deficiency compared to a U.S. population. In the Nigerian cohort, plasma concentrations of retinol were not correlated between mothers and cord blood (*r* = 0.05, *p* = 0.66).

### 3.3. Plasma Carotenoid Results

In contrast to plasma retinol concentrations, Nigerian maternal and cord concentrations of vitamin A-related compounds were significantly correlated for lutein + zeaxanthin, β-cryptoxanthin, α-carotene, and β-carotene. The maternal-cord correlations are shown in Table 3. While carotenoids were correlated in both populations, lycopene concentrations in the U.S. cohort were also statistically significant, and the correlations were stronger in the U.S. cohort. We also present a comparison of plasma concentrations of retinoids and carotenoids of the maternal populations (Table 4) and the infant populations (Table 5) in Nigeria and the U.S. In the maternal cohort, U.S. mothers had significantly higher plasma retinol concentrations, but lower levels of plasma carotenoids. Infants in the U.S. and Nigeria were born with similar plasma retinol concentrations, but significantly different carotenoid concentrations.

### 3.4. Socioeconomic Outcomes

In Nigerian dyads, cord concentrations in infants of unemployed mothers vs. employed were significantly lower for retinol (0.43 vs. 0.56 umol/L, *p* = 0.02), lutein + zeaxanthin (39.7 vs. 61.6 µg/L, *p* = 0.03), α-carotene (93.9 vs 224.4 µg/L, *p* = 0.01), β-cryptozanthin (31.3 vs. 52.2 µg/L, *p* = 0.02), and β-carotene (94.5 vs. 264.8 µg/L, *p* = 0.004). Cord retinol levels were also significantly lower for mothers with >12 years education vs. <12 years (0.47 vs. 0.58 umol/L, *p* = 0.02). There were no significant differences in maternal nutrient concentrations by employment status or education.

### 3.5. Infectious Disease Outcomes

In Nigerian dyads, statistically significant differences between maternal levels of lutein + zeaxanthin were present according to malaria status; women who had a diagnosis of malaria presented lower plasma concentrations compared to women without (173.0 vs. 237.8 µg/L for yes vs. no, respectively, *p* = 0.03), with similar results seen in their infants (28.4 vs. 50.8 µg/L for yes vs. no, respectively, *p* = 0.03). A trend toward similar results was also seen with a maternal diagnosis of HIV, with infants born to HIV+ mothers demonstrating a trend towards lower plasma lutein concentrations (23.7 vs. 46.6 µg/L, *p* = 0.06). Maternal plasma concentrations of α-carotene and β-carotene were higher in cases with a newborn evaluation for sepsis (α-carotene: 2,103.7 vs. 1,261 µg/L for yes vs. no sepsis, *p* = 0.05; β-carotene: 2848.7 vs. 1563.0 for yes vs. no sepsis, *p* = 0.05).

### 3.6. Delivery Outcomes

Statistically significant differences were also found between infant plasma lycopene levels and mode of delivery, with infants born vaginally having higher plasma lycopene concentrations than those delivered via Caesarean (87.8 vs. 47.9 respectively; *p* = 0.001). A similar effect was seen for infant concentrations of β-cryptoxanthin (46.5 vs. 19.1 µg/L for vaginal vs. Cesarean, *p* = 0.02). Additionally, a trend toward improved Apgar scores (≤7) at 5 min was also seen in infants with higher plasma lutein concentrations (46.7 vs. 26.7 µg/L for above vs. below 7, *p* = 0.06).

### 3.7. Growth Outcomes

In Nigerian dyads, infant plasma retinol concentrations were positively correlated with birth weight (*r* = 0.24, *p* = 0.04), while both maternal β-carotene and α-carotene concentrations showed an inverse correlation with infant birth weight (*r* = −0.22, *p* = 0.03 for β-carotene, *r* = −0.21, *p* = 0.04 for α-carotene). Infants who were born with a weight-for-age z score of -2 or lower had lower plasma retinol concentrations compared to those above (0.35 vs. 0.49 for above vs. below, *p* = 0.04). Conversely, mother of infants with a weight-for-age z score below −2 had higher plasma α-carotene (1926.9 vs. 1266.4 µg/L for below vs. above, *p* = 0.02) and β-carotene (2644.5 vs. 1565.7 µg/L for below vs. above, *p* = 0.04).

## 4. Discussion

Our study of retinoids and carotenoids in a developed vs. developing nation show a high prevalence of vitamin A deficiency among Nigerian mothers despite plasma concentrations of pro-vitamin A compounds that exceed those seen in a developed nation. Improvements in markers of socioeconomic status were associated with improvements in plasma concentrations of nutrients. Higher maternal carotenoid levels were associated with poorer outcomes, while higher cord carotenoid levels were associated with improved outcomes, possibly indicating the importance of the transfer from mother to infant.

Our findings of a high prevalence of vitamin A deficiency in this population are consistent with other reports of plasma retinol concentrations in pregnant women in developing countries. According to the WHO, VAD is a long-standing public health problem in sub-Saharan Africa [21]. Plasma vitamin A concentrations of ≤0.70 ug/L were reported in 48.5% of pregnant women in a 2011 study in an urban setting in Southeast Nigeria [26], although our recruitment in a prenatal clinic was able to capture both urban and rural Nigerian women. High rates of vitamin A deficiency during pregnancy are concerning for maternal health, as it is possible that mothers who fail to return to their pre-pregnancy retinol status before another pregnancy may end up having their retinol stores progressively depleted with each subsequent pregnancy. These trends are also problematic if vitamin A deficiency in the offspring is not corrected during early childhood, as 20–24% of child mortality from measles, diarrhea, and malaria and 3% of mortality associated with other infectious causes can be attributed to vitamin A deficiency [27]. Vitamin A deficiency in young children is a significant public health concern in Nigeria; a 2006 nationally representative survey of children under 5 years of age in Nigeria reported that rates of VAD ranges from 25–31% [28]. However, a recent 2018 report of 170 Southwestern Nigerian children under 5 years of age indicated that the rate of VAD was 5.3%, suggesting that there may be regional differences in rates of VAD, or the effectiveness of public health interventions in this area [29].

Very few studies have reported the carotenoid status of healthy pregnant women in sub-Saharan Africa for comparison to our population. A 2005 study of plasma antioxidant levels during pregnancy in women in Trujillo, Peru, reported that plasma concentration of α-carotene, β-carotene, lutein, and lycopene increased significantly from the first trimester of pregnancy to the third. Only β-crypoxanthin showed no change over time [30]. Compared to our study, the levels reported during the third trimester of pregnancy for the Peruvian women were higher in lutein (327 µg/L for the Peruvian women vs. 215 µg/L for the Nigerian women), but the Nigerian women in our study had higher concentrations of α-and β-carotene (1441 vs. 119 ug/L (α-carotene) and 1856 vs 134 ug/L (β-carotene)) for the Nigerian vs. Peruvian women. A study of pregnant women in the Netherlands yielded similar findings, with pregnant women in the Netherlands having similar lycopene and lutein levels to our Nigerian cohort, but concentrations of α- and β-carotene which were less than 10% of the Nigerian women [31]. The plasma concentration of the pregnant women in the Netherlands closely resembled the concentrations in the U.S. cohort reported in this study.

Studies of plasma carotenoids have been reported in populations of who are positive for HIV infection. A study of pregnant women with and without HIV infection in Malawi showed no significant differences in plasma carotenoid concentrations between HIV-positive and -negative women [32]. In contrast to our Nigerian cohort, lycopene and lutein were the predominant plasma carotenoids in the Malawi women [32], with lower α- and β-carotene concentrations than the Nigerian women. Another study evaluated plasma carotenoid concentrations in infants 14 weeks of age in relation to growth outcomes and mortality at one year, and found that infants with lower plasma carotenoids had an increased mortality rate [33]. The plasma concentrations of α-carotene, β-carotene, β-cryptoxanthin, and lycopene in the HIV infected infants ranged from 50 to 90% lower than the infants we report in this study; however, our samples were drawn at delivery and may not provide an adequate comparison to infant 14 weeks old. The HIV-infected infants were a more malnourished population, with 11.3% of the infants underweight and another 19% with growth stunting [33]. The Nigerian mothers in our study had significantly higher levels of plasma carotenoids but lower levels of retinol when compared to our U.S. populations, which may reflect differences in dietary intake. Fortification of foods with pre-formed vitamin A is common in the United States, as is access to animal products, including meat, eggs, and dairy. In general, a rural Nigerian diet is much more plant-based and relies on the conversion of pro-vitamin A compounds into retinol. In addition, consumption of red palm oil is common in pregnant Nigerian women, which is an excellent source of α- and β-carotenes. Supplementation with red palm oils has been shown to increase serum carotenoid levels, but not retinol levels, in other populations of African women [34]. Results from the Norwegian Mother and Child Cohort Study confirms that plasma concentrations of pro-vitamin A carotenoids are a strict function of intakes of fruits and vegetables [35,36]. Intakes of carotenoids in pregnancy have been shown to vary widely as depend on the population in question [15], and carotenoid intake has also been shown to be lower in smokers and younger women and dependent on season, which may be relevant to a rural Nigerian population [15].

The nutrition status of a population can depend to a great extent on its socioeconomic status. Earlier studies in a Nigerian urban setting showed no association between household characteristics such as occupation and household size with plasma retinol concentrations; however, education had a positive impact [26]. While our study did not find an effect of education or employment on maternal plasma levels, the infants of these mothers did have higher plasma nutrient concentrations. Increased income and education allow for greater flexibility in food choices, and employed mothers may be more likely to be urban residents and have access to a wider range of food products. However, maternal employment could also lead to less time for shopping and cooking, and preparing the energy-intensive staples of traditional diets. Studies in pregnant women in other developing nations have shown that plasma levels of carotenoids increase as education level increases [30], and have documented a shift in intakes of carotenoid-foods between pregnant women of higher and lower educational status, with higher education leading to increased intakes [37].

This study reports that lower plasma retinol concentrations in infants were associated with poor growth parameters. Observational studies have reported that low maternal plasma retinol concentrations are associated with intrauterine growth restriction, although these results were reported with HIV-infected women [3]. The U.S. cohort reported in this study did show that mothers with insufficient concentrations of vitamin A delivered more children with low birth weight (<2500 g), while more infants with deficient cord concentrations of retinol (≤0.7 µmol/L) were born small for gestational age [6]. A meta-analysis that evaluated the effects of vitamin A supplementation during pregnancy on multiple outcomes related to maternal, perinatal, and infant health showed that the overall effects of vitamin A on small for gestational age (SGA) and very low birth weight (<2.0 kg) were null, and that for low birth weight (<2.5 kg) was null but with a trend towards significance [3]. In contrast to our study, a case-control study conducted in Canada that assessed correlations between mid-pregnancy antioxidants concentrations and SGA found an increased retinol concentration as being associated with a significantly increased risk of both mild and severe SGA [38]. Interestingly, an increased carotenoid concentration was associated with a significantly reduced risk of both mild and severe SGA in this same study [38]. Other studies have shown positive associations between maternal carotenoid status and infant growth, specifically lycopene [39]. As far as we know, our study is the first to report that maternal levels of α- and β-carotenoids are higher in mothers of growth-restricted infants. There are several possible explanations for this finding. It is possible that high maternal levels of carotenoids may represent placental dysfunction that leads to defective transport of nutrients to the developing fetus. Studies of retinol have shown that amniotic fluid levels of retinol are higher in normal pregnancies than those with complications such as pre-elecampsia. Taken in conjunction with other studies that have shown that retinol concentrations are higher in mothers with growth restricted infants [38,40], higher maternal micronutrient levels may be a marker of poor placental function and could signify lower levels of nutrients reaching the fetus. As carotenoids stimulate growth directly at a cellular level [41], this may be of causal importance for fetal growth. Similar inferences could be made for our findings of higher α- and β-carotene levels in infants with a newborn evaluation for sepsis.

An interesting finding in this study was a trend between maternal lutein concentrations and infant Apgar scores at 5 min. Lutein is the dominant carotenoid found in the infant brain, especially in the neocortex and in the neural retina [42,43,44]. During the first month postpartum, lutein remains relatively elevated in the human milk, demonstrating a potential important role for the infant [45]. Preterm infants fed with lutein-supplemented formula seem to develop less severe retinopathy of prematurity [46]. Taken together, these findings make it exciting to speculate on the potential for lutein as a target to improve neurological outcomes.

Our study has several limitations. Our sample size is small for a prevalence estimate, and we have a low occurrence of infectious outcomes such as malaria. We did not have measurements of zinc status available, and low plasma retinol concentrations may result from inadequate plasma zinc, which is required for a normal rate of synthesis of retinol binding protein. Plasma retinol concentration may also be low during inflammation or infection because of transient decreases in the concentrations of the negative acute phase proteins, retinol binding protein, and transthyretin, even when liver retinol is adequate. Our small sample size made it difficult to assess for the many confounders that could be associated with fetal growth and outcomes; however, our choices of gestational age and smoking are likely to have a large confounding effect.

## 5. Conclusions

Despite plasma concentrations of pro-vitamin A carotenoids which are higher than those reported in other populations, pregnant Nigerian women have a high prevalence of vitamin A deficiency.

## Figures and Tables

**Table 1:** Demographic characteristics of the participants.

	United States Maternal Population		Nigerian Maternal Population		*p* Value
	N	Mean (SD)	N	Mean (SD)	
**Mean age** (years)	179	28.6 (5.6)	98	31.1 (4.7)	0.0002
**Mean Pre-pregnancy BMI** (kg/m^2^)	105	27.1 (6.6)	99	31.1 (4.2)	<0.001
**Gestational Age at Delivery** (weeks of gestation)	179	38.2 (3.1)	99	38.4 (2.4)	0.49
**Infant Birth Anthropometrics**:					
Birth Weight (g)	179	3145.2 (735.1)	99	3086.2 (479.1)	0.42
Birth Length (cm)	179	48.5 (4.7)	99	49.3 (3.8)	0.14
Birth head circumference (cm)	179	33.6 (2.8)	99	34.4 (2.4)	0.01
	*N* (%)		*N* (%)		
**Mode of delivery**					
Vaginal Delivery	119 (66)		67 (68)		0.89
Caesarian Section	60 (34)		32 (32)		
**Gender**					
Male (%)	90 (50)		50 (51)		1.0
Female (%)	89 (50)		49 (49)		
**Smoking status**					
Current Smokers	26 (15)		1 (1)		<0.001
Former/never Smokers	152 (85)		97 (99)		
**Malaria (Maternal Dx)**	N/A				
No			15 (15.1)		
Yes			5 (5.0)		
Unknown			79 (79.8)		
**Maternal HIV status**	N/A				
Negative			94 (95)		
Positive			5 (5)		
**Newborn sepsis evaluation**	N/A				
No			90 (90.9)		
Yes			9 (9.1)		

Dx: diagnosis; BMI: Body Mass Index.

**Table 2:** Retinol Status of Mothers and Infants in the U.S. and Nigeria.

Infants	U.S. *N* (%)	Nigeria *N* (%)
Severely deficient (below 0.35 µmol/L)	14 (7.6)	11 (14.8)
Deficient (0.35–0.70 µmol/L)	138 (72.8)	50 (67.6)
Insufficient (0.70–1.05 µmol/L)	34 (17.9)	12 (16.2)
Adequate (above 1.05 µmol/L)	3 (1.7)	1 (1.4)
Mothers		
Severely deficient (below 0.35 µmol/L)	1 (0.7)	0
Deficient (0.35–0.70 µmol/L)	18 (9.3)	30 (35.5)
Insufficient (0.70–1.05 µmol/L)	78 (41.4)	35 (40.2)
Adequate (above 1.05 µmol/L)	92 (48.6)	22 (25.3)

**Table 3:** Correlations of maternal and cord concentrations of vitamin A related compounds in Nigerian and U.S. cohorts.

	Nigerian Cohort		US Cohort	
Plasma Level	Correlation Coefficient (*r*)	*p*-Value	Correlation Coefficient (*r*)	*p*-Value
Lutein + zeaxanthin	0.44	0.0001	0.45	˂0.0001
β-cryptoxanthin	0.29	0.01	0.64	˂0.0001
Lycopene	0.18	0.15	0.30	˂0.0001
α-carotene	0.35	0.003	0.70	˂0.0001
β-carotene	0.43	0.0002	0.65	˂0.0001

**Table 4:** Comparison of median plasma concentrations of retinoids and carotenoids between Nigerian and U.S. mothers.

Plasma Nutrient Median (IQR)	Nigeria	US	*p*-Value
Retinol (µmol/L)	0.81 (0.43)	1.06 (0.41)	˂0.001
Lutein+zeaxanthin (µg/L)	218.3 (83.7)	183.9 (106.9)	0.03
Lycopene (µg/L)	510.2 (269.4)	422.0 (289.0)	0.03
β-cryptoxanthin (µg/L)	187.2 (86.7)	91.2 (77.8)	˂0.001
β-carotene (µg/L)	1,623.8 (1,763.7)	151.3 (184.2)	˂0.001
α-carotene (µg/L)	1,284.7 (1,047.2)	29.4 (53.1)	˂0.001

**Table 5:** Comparison of plasma concentrations of retinoids and carotenoids between Nigerian and U.S. infants.

Plasma Nutrient Median (IntraQuartile Range)	Nigeria	US	*p*-Value
Retinol (µmol/L)	0.49 (0.19)	0.52 (0.23)	0.12
Lutein+zeaxanthin (µg/L)	45.2 (25.8)	27.8 (17.1)	˂0.001
Lycopene (µg/L)	61.4 (79.5)	16.3 (13.0)	˂0.001
β-cryptoxanthin (µg/L)	35.7 (28.9)	8.9 (7.9)	˂0.001
β-carotene(µg/L)	105.3 (102.2)	9.3 (10.6)	˂0.001
α-carotene (µg/L)	103.9 (85.7)	3.4 (4.4)	˂0.001

## References

[1] Azais-Braesco V., Pascal G. (2000). Vitamin A in pregnancy: Requirements and safety limits. Am. J. Clin. Nutr..

[2] Stevens G.A., Bennett J.E., Hennocq Q., Lu Y., De-Regil L.M., Rogers L., Danaei G., Li G., White R.A., Flaxman S.R. (2015). Trends and mortality effects of vitamin A deficiency in children in 138 low-income and middle-income countries between 1991 and 2013: A pooled analysis of population-based surveys. Lancet Glob. Health.

[3] Thorne-Lyman A.L., Fawzi W.W. (2012). Vitamin A and carotenoids during pregnancy and maternal, neonatal and infant health outcomes: A systematic review and meta-analysis. Paediatr. Perinat. Epidemiol..

[4] McCauley M.E., van den Broek N., Dou L., Othman M. (2015). Vitamin A supplementation during pregnancy for maternal and newborn outcomes. Cochrane Database Syst. Rev..

[5] Radhika M.S., Bhaskaram P., Balakrishna N., Ramalakshmi B.A., Devi S., Kumar B.S. (2002). Effects of vitamin A deficiency during pregnancy on maternal and child health. BJOG.

[6] Hanson C., Lyden E., Abresch C., Anderson-Berry A. (2016). Plasma retinol concentrations, race, and socioeconomic status in of women of childbearing age in the united states. Nutrients.

[7] Food and Nutrition Board, Institute of Medicine (2001). Dietary Reference Intakes for Vitamin A, Vitamin K, Arsenic, Boron, Chromium, Copper, Iodine, Manganese, Molybdenum, Nickel, Silion, Vandadium, Zinc.

[8] Semba R.D., Miotti P.G., Chiphangwi J.D., Sah A.J., Canner J.K., Dallabetta G.A., Hoover D.R., Canner MHS J.K., Aj Saah M.D. (1994). Maternal vitamin A deficiency and mother-to-child transmission of HIV-1. Lancet.

[9] World Health Organization and Food and Agriculture Organization (2004). Vitamin and Mineral Requirements in Human Nutriion: Report of a Joint FAO/WHO Expert Consulation, Bangkok, Thailand, 21–30 September 1998.

[10] Sommer A., Vyas K.S. (2012). A global clinical view on vitamin A and carotenoids. Am. J. Clin. Nutr..

[11] Giampietri M., Lorenzoni F., Moscuzza F., Boldrini A., Ghirri P. (2016). Lutein and neurodevelopment in preterm infants. Front. Neurosci..

[12] Food and Nutrition Board, Institute of Medicine (2000). Dietary Reference Intakes for Vitamin C, Vitamin E, Selenium, and Carotenoids.

[13] Bouch S., Harding R., O’Reilly M., Wood L.G., Sozo F. (2016). Impact of dietary tomato juice on changes in pulmonary oxidative stress, inflammation and structure induced by neonatal hyperoxia in mice (mus musculus). PLoS ONE.

[14] Erdman J.W., Ford N.A., Lindshield B.L. (2009). Are the health attributes of lycopene related to its antioxidant function?. Arch. Biochem. Biophys..

[15] Zielinska M.A., Wesolowska A., Pawlus B., Hamulka J. (2017). Health effects of carotenoids during pregnancy and lactation. Nutrients.

[16] Christian P., West K.P., Khatry S.K., Katz J., LeClerq S.C., Kimbrough-Pradhan E., Dali S.M., Shrestha S.R. (2000). Vitamin A or beta-carotene supplementation reduces symptoms of illness in pregnant and lactating nepali women. J. Nutr..

[17] Christian P., West K.P., Khatry S.K., Katz J., LeClerq S., Pradhan E.K., Shrestha S.R. (1998). Vitamin A or beta-carotene supplementation reduces but does not eliminate maternal night blindness in nepal. J. Nutr..

[18] West K.P., Christian P., Labrique A.B., Rashid M., Shamim A.A., Klemm R.D.W., Massie A.B., Mehra S., Schulze K.J., Ali H. (2011). Effects of vitamin A or beta carotene supplementation on pregnancy-related mortality and infant mortality in rural bangladesh: A cluster randomized trial. JAMA.

[19] West K.P., Katz J., Khatry S.K., Rashid M., LeClerq S.C., Pradhan E.K., Shrestha S.R., Connor P.B., Dali S.M., Christian P. (1999). Double blind, cluster randomised trial of low dose supplementation with vitamin A or beta carotene on mortality related to pregnancy in nepal. the NNIPS-2 study group. BMJ.

[20] El-Sohemy A., Baylin A., Kabagambe E., Ascherio A., Spiegelman D., Campos H. (2002). Individual carotenoid concentrations in adipose tissue and plasma as biomarkers of dietary intake. Am. J. Clin. Nutr..

[21] World Health Organization (2009). Global prevalence of vitamin A deficiency in populations at risk 1995–2005: WHO global database on vitamin A deficiency. http://apps.who.int/iris/handle/10665/44110.

[22] Sivakumar B., Panth M., Shatrugna V., Raman L. (1997). Vitamin A requirements assessed by plasma response to supplementation during pregnancy. Int. J. Vitam. Nutr. Res..

[23] Tyson J.E., Wright L.L., Oh W., Kennedy K.A., Mele L., Ehrenkranz R.A., Stoll B.J., Lemons J.A., Stevenson D.K., Bauer C.R. (1999). Vitamin A supplementation for extremely-low-birth-weight infants. national institute of child health and human development neonatal research network. New Engl. J. Med..

[24] Biswas A.B., Mitra N.K., Chakraborty I., Basu S., Kumar S. (2000). Evaluation of vitamin A status during pregnancy. J. Indian Med. Assoc..

[25] Hanson C., Schumacher M., Lyden E., Furtado J., Van Ormer M., McGinn E., Rilett K., Cave C., Johnson R., Weishaar K. (2017). Status of vitamin A and related compounds and clinical outcomes in maternal-infant pairs in the midwestern united states. Ann. Nutr. Metab..

[26] Williams I.O., Essien E.U., Eka O.U. (2011). Socioeconomic factors and vitamin a status of pregnant women in calabar urban, southeastern nigeria. Matern. Child. Health J..

[27] Humphrey J., West K., Sommer A. Vitamin A deficiency and attributable mortality among under-5-year-olds. http://apps.who.int/iris/bitstream/handle/10665/49301/bulletin_1992_70%282%29_225-232.pdf?sequence=1&isAllowed=y.Updated20172018.

[28] Maziya-Dixon B.B., Akinyele I.O., Sanusi R.A., Oguntona T.E., Nokoe S.K., Harris E.W. (2006). Vitamin A deficiency is prevalent in children less than 5 y of age in nigeria. J. Nutr..

[29] Abolurin O.O., Adegbola A.J., Oyelami O.A., Adegoke S.A., Bolaji O.O. (2018). Prevalence of vitamin A deficiency among under-five children in south-western nigeria. Niger Postgrad. Med. J..

[30] Horton D.K., Adetona O., Aguilar-Villalobos M., Cassidy B.E., Pfeiffer C.M., Schleicher R.L., Caldwell K.L., Needham L.L., Rathbun S.L., Vena J.E. (2013). Changes in the concentrations of biochemical indicators of diet and nutritional status of pregnant women across pregnancy trimesters in trujillo, peru, 2004–2005. Nutr. J..

[31] Oostenbrug G.S., Mensink R.P., van Houwelingen A.C., Al M.D.M., Hornstra G. (1998). Pregnancy-induced hypertension: Maternal and neonatal plasma lipid-soluble antioxidant levels and its relationship with fatty acid unsaturation. Eur. J. Clin. Nutr..

[32] Lan Y., Kumwenda N., Taha T.E., Chiphangwi J.D., Miotti P.G., Mtimavalye L., Broadhead R., van der Hoeven L., Hoover D.R., Semba R.D. (1999). Carotenoid status of pregnant women with and without HIV infection in malawi. East. Afr. Med. J..

[33] Lietz G., Henry C., Mulokozi G., Mugyabuso J.K.L., Ballart A., Ndossi G.D., Lorri W., Tomkins A. (2001). Comparison of the effects of supplemental red palm oil and sunflower oil on maternal vitamin A status. Am. J. Clin. Nutr..

[34] Melikian G., Mmiro F., Ndugwa C., Perry R., Jackson J.B., Garrett E., Tielsch J., Semba R.D. (2001). Relation of vitamin a and carotenoid status to growth failure and mortality among ugandan infants with human immunodeficiency virus. Nutrition.

[35] Brantsæter A.L., Haugen M., Hagve T.-A., Aksnes L., Rasmussen S.E., Julshamn K., Alexander J., Meltzer H.M. (2007). Self-reported dietary supplement use is confirmed by biological markers in the norwegian mother and child cohort study (moba). Ann. Nutr. Metab..

[36] Brantsæter A.L., Haugen M., Rasmussen S.E., Alexander J., Samuelsen S.O., Meltzer H.M. (2007). Urine flavonoids and plasma carotenoids in the validation of fruit, vegetable and tea intake during pregnancy in the norwegian mother and child cohort study (moba). Public Heal. Nutr..

[37] Freisling H., Elmadfa I., Gall I. (2006). The effect of socioeconomic status on dietary intake, physical activity and body mass index in austrian pregnant women. J. Hum. Nutr. Diet..

[38] Cohen J.M., Kahn S.R., Platt R.W., Basso O., Evans R.W., Kramer M.S. (2015). Small-for-gestational-age birth and maternal plasma antioxidant levels in mid-gestation: A nested case-control study. BJOG.

[39] Hanson C., Lyden E., Furtado J., Ormer M.V., White K., Overby N., Anderson-Berry A. (2018). Plasma lycopene concentrations and associations with clinical outcomes in a cohort of maternal-infant dyads. Nutrients.

[40] Ortega-Senovilla H., Alvino G., Taricco E., Cetin I., Herrera E. (2010). Enhanced circulating retinol and non-esterified fatty acids in pregnancies complicated with intrauterine growth restriction. Clin. Sci..

[41] Mathews F. (1996). Antioxidant nutrients in pregnancy: A systematic review of the literature. Nutr. Res. Rev..

[42] Costa S., Giannantonio C., Romagnoli C., Barone G., Gervasoni J., Perri A., Zecca E. (2015). Lutein and zeaxanthin concentrations in formula and human milk samples from italian mothers. Eur. J. Clin. Nutr..

[43] Vishwanathan R., Kuchan M.J., Sen S., Johnson E.J. (2014). Lutein and preterm infants with decreased concentrations of brain carotenoids. J. Pediatr. Gastroenterol. Nutr..

[44] Vishwanathan R., Neuringer M., Snodderly D.M., Schalch W., Johnson E.J. (2013). Macular lutein and zeaxanthin are related to brain lutein and zeaxanthin in primates. Nutr. Neurosci..

[45] Gossage C.P., Deyhim M., Yamini S., Douglass L.W., Moser-Veillon P.B. (2002). Carotenoid composition of human milk during the first month postpartum and the response to beta-carotene supplementation. Am. J. Clin. Nutr..

[46] Rubin L.P., Chan G.M., Barrett-Reis B.M., Fulton A.B., Hansen R.M., Ashmeade T.L., Oliver J.S., Mackey A.D., Dimmit R.A., Hartmann E.E. (2011). Effect of carotenoid supplementation on plasma carotenoids, inflammation and visual development in preterm infants. J. Perinatol..

