# Erratum: Status of Retinoids and Carotenoids and Associations with Clinical Outcomes in Maternal-Infant Pairs in Nigeria. *Nutrients* 2018, *10*, 1286

**DOI:** 10.3390/nu11020396

**Published:** 2019-02-13

**Authors:** Corrine Hanson, Elizabeth Lyden, Ann Anderson-Berry, Nicholas Kocmich, Amy Rezac, Shirley Delair, Jeremy Furtado, Matthew Van Ormer, N. Izevbigie, EK Olateju, Godwin O. Akaba, EA Anigilaje, Thairu Yunusa, Stephen Obaro

**Affiliations:** 1 College of Allied Health Professions Medical Nutrition Education, University of Nebraska Medical Center, Omaha, NE 68198-4045, USA; 2 College of Public Health, University of Nebraska Medical Center, Omaha, NE 68198-4375, USA; elyden@unmc.edu; 3 Pediatrics 981205 Nebraska Medical Center, University of Nebraska Medical Center, Omaha, NE 68198-1205, USA; alanders@unmc.edu (A.A.-B.); nicholas.kocmich@unmc.edu (N.K.); amy.rezac@unmc.edu (A.R.); shirley.delair@unmc.edu (S.D.); matthew.vanormer@unmc.edu (M.V.O.); stephen.obaro@unmc.edu (S.O.); 4 Department of Nutrition, Harvard School of Public Health 655 Huntington Avenue, Boston, MA 02215, USA; jfurtado@hsph.harvard.edu; 5 University of Abuja Teaching Hospital Gwagwalada-Zuba, Gwagwalada P.M.B. 228, Nigeria; sakharemog@yahoo.co.uk (N.I.); oeyinade@yahoo.com (E.K.O.); docakabago@yahoo.com (G.O.A.); naijanannytales@yahoo.com (E.A.A.); samhaamal200@gmail.com (T.Y.)

Due to a mistake during the production process, there were spelling errors in four of the author names in the original published version [1]. The Nutrients Editorial Office therefore wishes to make the following corrections to the paper.

The names of the ninth author, “NI Izevbigie”, the tenth author, “ED Olateju”, the eleventh author, “GA Adaba”, and the thirteenth author, “Tahiru Tahiru”, have been changed to “N. Izevbigie”, “EK Olateju”, “Godwin O. Akaba” and “Thairu Yunusa”, respectively. The thirteenth author’s email address “tahiroshma@yahoo.com” has been changed into “samhaamal200@gmail.com”.

We would like to apologize for any inconvenience caused to the authors and readers by this mistake. We will update the article, and the original version will remain available on the article webpage.
